# Extracellular Polysaccharides from *Ganoderma lucidum* as a Functional Ingredient: Improving the Technological Quality and Bioactive Properties of Corn Noodles

**DOI:** 10.3390/foods15142463

**Published:** 2026-07-11

**Authors:** Jinshan Wu, Aoran Guo, Yujia Ni, Dali Zhang, Huimin Liu

**Affiliations:** 1 College of Food Science and Engineering, Jilin Agricultural University, Changchun 130118, China; wujs0709@163.com (J.W.); 15633966010@163.com (A.G.); nyj66926@163.com (Y.N.); 2 National Engineering Research Center of Wheat and Corn Further Processing, Changchun 130118, China

**Keywords:** corn noodles, *Ganoderma lucidum* extracellular polysaccharides, technological quality, bioactive properties

## Abstract

Corn noodles, as a staple food, suffer from poor processing quality and high starch digestibility due to the lack of a gluten network. Magnetically treated *Ganoderma lucidum* extracellular polysaccharides (MEPSs) possess excellent water-binding and gel-forming properties. This study aimed to investigate the effects of MEPSs at concentrations of 0%, 0.2%, 0.4%, 0.6%, 0.8%, and 1.0% on the technological quality and functional properties of corn noodles and to elucidate the underlying mechanisms through rheological, microstructural, and molecular analyses. The results showed that 0.6% MEPSs was the optimal concentration. Adding MEPSs enhanced dough viscoelasticity and water-holding capacity and induced a compact, continuous gel network with increased short-range molecular order and disulfide crosslinks. Consequently, the cooking loss and breaking rate of corn noodles decreased by 24.69% and 46.65%, respectively, while the hardness and springiness improved and cohesiveness was reduced. Functionally, adding 0.6% MEPSs inhibited α-amylase and α-glucosidase, slowing starch hydrolysis and reducing the estimated glycemic index from 76.4 to 72.3. It also significantly enhanced antioxidant activities, including DPPH, ABTS, and hydroxyl radical scavenging, as well as FRAP. In conclusion, MEPS is a natural multifunctional ingredient that simultaneously improves technological quality and confers hypoglycemic and antioxidant benefits to corn noodles, providing an efficient strategy for developing healthier staple foods.

## 1. Introduction

Corn is one of the most important cereal crops in the world [1]. Corn noodles are popular among consumers due to their unique flavor and rich content of dietary fiber, vitamins, and minerals [2]. However, their technological quality is poor. Different from wheat flour, corn flour contains little gluten protein and thus cannot form a continuous and stable network during dough development. It forces starch to become the main structural component of the noodle matrix, but corn starch lacks sufficient swelling power and water-binding capacity to form a strong gel network during cooking [3]. Neither the sparse gluten protein nor the starch gel alone can provide adequate structural support. Consequently, corn noodles exhibit a high breakage rate, high cooking loss, and poor texture during processing and consumption [4], which severely limits their development as a mainstream staple food.

Various strategies have been explored to improve the quality of corn noodles. The addition of hydrocolloids (such as xanthan gum, sodium alginate and carrageenan) is a common method to improve the quality of low-gluten noodles. Studies have shown that hydrocolloids can significantly improve the textural properties and cooking quality of corn noodles by enhancing the water-holding capacity and viscoelasticity of dough [5]. Vital gluten is also commonly used as a dough improver to enhance the quality of corn noodles [6]. In addition, enzyme preparations and pregelatinized starch have been used to strengthen the network structure of noodles [7]. However, most of these modification strategies focus on the improvement of processing quality. Less attention has been paid to the nutritional function and health benefits of noodles.

Edible mushroom polysaccharides have attracted considerable research interest due to their excellent safety, biodegradability, and diverse bioactivities [8,9]. Studies have shown that mushroom polysaccharides can interact with starch molecules through hydrogen bonding and physical entanglement. These interactions regulate starch gelatinization, gelation, and digestive properties [10]. For instance, *Lyophyllum decastes* polysaccharides could inhibit starch gelatinization, reduce peak viscosity, increase resistant-starch content, and slow down starch hydrolysis through direct inhibition of α-glucosidase and physical encapsulation of starch granules [11]. Similarly, *Lentinus edodes* polysaccharides enhanced the pasting temperature, storage modulus and loss modulus of dough, changed the proportion of protein secondary structure, and increased the amount of resistant starch [12]. These studies confirm the dual potential of edible mushroom polysaccharides in improving processing quality and regulating starch digestion.

Among them, *Ganoderma lucidum* is a well-known medicinal and edible mushroom. Its extracellular polysaccharides have many physiological activities such as anti-oxidation, immune regulation and hypoglycemic activity [13,14]. The chemical composition and structural characteristics of *Ganoderma lucidum* polysaccharides directly affect their functional performance. However, conventional fermentation often yields limited amounts of *Ganoderma lucidum* extracellular polysaccharides with relatively homogeneous structures and moderate bioactivities. Therefore, researchers have optimized the fermentation strategy to regulate the yield and structure of extracellular polysaccharides from *Ganoderma lucidum*. Recently, physical field-assisted fermentation strategies have been shown to effectively improve the yield and functional properties of fungal polysaccharides. Static magnetic-field-assisted fermentation is a mild and green physical elicitation approach that has shown positive effects of improving microbial polysaccharide production and structural characteristics. Studies have demonstrated that static magnetic-field treatment can affect cell membrane permeability and energy metabolism of microorganisms [15]. It can further regulate the activities of key enzymes involved in polysaccharide biosynthesis pathways. Our previous research found that the extracellular polysaccharides of *Ganoderma lucidum* obtained through static magnetic-field-assisted fermentation exhibited higher yield and bioactivities than those produced by traditional fermented polysaccharides.

However, to date, no studies have investigated the incorporation of magnetically treated *Ganoderma lucidum* extracellular polysaccharides into corn noodles, nor have they systematically evaluated the simultaneous improvement of processing quality and functional properties. Therefore, the present study aimed to investigate the effects of MEPS on the technological quality and bioactive properties of corn noodles.

## 2. Materials and Methods

### 2.1. Materials and Regrents

Corn flour (Jinzhou Rongqiang Grains Co., Ltd., Jinzhou, China), gluten prion powder (Ruirente Biotechnology Co., Ltd., Zhengzhou, China), a BCA Protein Concentration Assay Kit (Beyotime Biotechnology Co., Ltd., Nantong, China), a Free-Radical-Scavenging-Capacity Assay Kit (Shanghai yuanye Bio-Technology Co., Ltd., Shanghai, China), Amyloglucosidase and α-Amylase (Sigma-Aldrich (Shanghai) Trading Co., Ltd., St. Louis, MO, USA) were purchased. All reagents were of analytical grade.

### 2.2. Preparation of Magnetism-Assisted Fermented Ganoderma lucidum Extracellular Polysaccharides (MEPSs) and Characterization of Main Components

The activated *Ganoderma lucidum* strain was inoculated into a liquid medium. The culture was then incubated at 150 r/min and 28 °C for 8 days. After 2 days of cultivation, the culture flask was transferred to a magnetic-field shaking incubator (MFI-L1 magnetic-field oscillation light incubator, INDUC Scientific Co., Ltd., Wuxi, China). It was further incubated under a magnetic field of 8 mT for 4 h. Subsequently, the flask was transferred back to a regular incubator (Stab S2 Full-Temperature Shaker Incubator, RADOBIO SCIENTIFIC Co., Ltd., Shanghai, China) for the remaining culture period. After the liquid fermentation was completed, the mycelia were removed by filtration, and the fermentation broth was retained. The supernatant was subjected to enzyme inactivation, concentration, alcohol precipitation, protein removal using the Sevage method, and dialysis. Finally, the MEPSs were obtained after freeze-drying (LGE-50E vacuum freeze dryer, Foring Technology Development Co., Ltd., Beijing, China).

The total sugar content of MEPS was determined using the phenol–sulfuric acid method [16]. The protein content of MEPS was measured using a BCA protein assay kit. The uronic acid content of MEPS was determined using the sulfuric acid–carbazole method [17].

### 2.3. Preparation of Corn Noodles with Different Proportions of MEPS

Corn flour, vital gluten and salt were prepared in a mixed powder. Different amounts of MEPS were dissolved in purified water, respectively. The mixed powder and the MEPS solutions were placed in a pin-type dough mixer and mixed for 3 min. The resulting dough pieces were passed through a noodle machine for 25 sheeting passes (with a roll gap of 2.0 mm) and corn noodles were then prepared. Corn noodles are named according to the amount of MEPS added, as shown in Table 1.

### 2.4. Determination of Gelatinization Characteristics

The gelatinization characteristics of corn flour with different proportions of MEPS were measured using a rapid visco analyzer (RVA) (3JK-1 Rapid Viscosity Analyzer, Perten Instruments Co., Ltd., Stockholm, Sweden). An aluminum canister containing mixed powder (3 g) and 25 mL of distilled water was placed into the analyzer and then fully stirred for determination. The measurement procedure was based on the method described by Yaqoob et al. [18]. The software model is TCW3.

### 2.5. Determination of Rheological Properties

The effect of different MEPS proportions on the storage modulus (G’), loss modulus (G’’), and loss factor (tanδ) of corn dough was measured using a rheometer (MCR302 rheometer, ANTON PAAR TRADING Co., Ltd., Graz, Austria). According to the sample preparation steps, the raw materials are made into dough, then cut into disks with a diameter of 40 mm. Each sample was placed on a rheometer sample stage, compressed to a 1.2 mm gap, and trimmed along the sample edges for measurement. The measurement method was slightly modified based on Jiang et al.’s approach [19], performing dynamic frequency scanning (0.1–100 rad/s) at 25 °C with 0.5% strain (linear viscoelastic region), recording G′, G’’ and tanδ.

### 2.6. Analysis of Water Distribution

The effect of different MEPS proportions on the water distribution of corn dough was measured using a low-field nuclear magnetic resonance (LF-NMR) analyzer (MR23-040V-I Low-Field Magnetic Resonance Spectrometer, Shanghai Niumag Electronic Technology Co., Ltd., Shanghai, China). Fresh sheeted dough (2 g) was placed into a 25 mm NMR sample tube for measurement. The measurement parameters were set as follows: waiting time (TW) = 1400 ms, number of echoes (NECH) = 1500, echo time (TE) = 0.30 ms, and number of scans (NS) = 5.

### 2.7. Determination of Steaming and Boiling Properties

Thirty fresh noodles were taken and weighed. The weight was recorded as *M*_1_. The noodles were cooked in 500 mL of boiling water for 3 min. After cooking, the noodles were removed and cooled in cold water for 30 s. The cooled noodles were taken out and the number of broken noodles was counted and recorded as *n*. Then, a kitchen paper towel was used to remove the surface moisture from the cooked noodles. The cooked noodles were weighed again, and the weight was recorded as *M*_2_. The cooked noodles were placed in a glass dish and dried in an oven to constant weight. The weight of the dried noodles was recorded as *M*_3_. A rapid moisture analyzer was used to determine the moisture content of the cooked noodles, which was recorded as *w*. The cooking water absorption, cooking loss, and breakage rate were calculated according to the following formulas.

(1) $$\mathrm{Cooking}\;\mathrm{loss}\;\left(\%\right)=\frac{M_{1}\left(1\;-\;w\right)\;-\;M_{3}}{M_{1}\left(1\;-\;w\right)}\;\times \;100\%\;$$

(2) $$\mathrm{Cooking}\;\mathrm{water}\;\mathrm{absorption}\;\left(\%\right)=\frac{M_{2}\;-\;M_{1}}{M_{1}}\;\times \;100\%$$

(3) $$\mathrm{Breakage}\;\mathrm{rate}\;\left(\%\right)=\frac{\;30\;-\;n}{30}\;\times \;100\%$$

### 2.8. Determination of Textural Properties

The effects of different MEPS proportions on the hardness, springiness, cohesiveness, and resilience of corn noodles were measured using a texture analyzer (TA-XT plus Stable Micro Systems Texture Analyser, Stable Micro Systems Co., Ltd., Godalming, Surrey, Britain) equipped with a P/36R probe. The noodle sample was placed on the test platform. The starting gap was set to 10 mm. The pre-test, test, and post-test speeds were all 1 mm/s. The trigger force was 5.00 g. The compression strain was set to 75%. The software model is TEE32VB v6.1.11.0.

### 2.9. Determination of Tensile Properties

The effects of different MEPS proportions on the extension distance and breaking force of corn noodles were measured using a texture analyzer equipped with an A/SPR probe. Measurement parameters refer to the research by Han et al. [20], as below: The pre-test, test, and post-test speeds were set to 1 mm/s, 1 mm/s, and 10 mm/s, respectively. The trigger force was 0.5 g. A tensile hook was used to pull the noodle upward until it broke. The tensile curve of the noodle was thus obtained.

### 2.10. Determination of Digestibility In Vitro

In vitro digestion was performed according to the method described by Tian et al. [21]. The glucose content in the digestion solution was measured using the 3,5-dinitrosalicylic acid (DNS) method. The starch hydrolysis rate was then calculated. The area under the hydrolysis curve (AUC) was calculated. The hydrolysis index (HI) was determined based on the AUC. The eGI of the corn noodles was calculated using the following formula:

(4) $$HI=\frac{{AUC}_{sample}}{{AUC}_{white\;bread}}\;\times \;100\;$$

(5) eGI=39.71+HI × 0.549 

### 2.11. Determination of Antioxidant Capacity

The cooked corn noodles were freeze-dried and ground into a powder. Aqueous ethanol was used to extract the active components from the corn noodle powder. According to the instructions for DPPH, ABTS, hydroxyl radical-scavenging-ability kits, and FRAP kits, the corresponding solvents were used to extract, centrifuge, and perform other pretreatments of the corn noodle powder, followed by antioxidant activity measurement following the steps.

### 2.12. Determination of Enzyme Inhibition Activity

The cooked corn noodles were freeze-dried and ground into a powder. Aqueous ethanol was used to extract the active components from the powder. The solvent was removed by rotary evaporation. The α-glucosidase and α-amylase inhibitory activities were then measured according to the method described by Ma et al. [22]. Acarbose was used as a positive control.

### 2.13. Observation of Microstructure

A small amount of freeze-dried corn noodle powder was placed onto conductive tape. The sample was then sputter-coated with gold. The microstructure of the corn noodles was observed using a scanning electron microscope (SEM) (MERLIN Compact, Carl Zeiss Inc., Jena, Germany) at an accelerating voltage of 5 kV.

### 2.14. Analysis of Short-Range Ordered Structure of Corn Noodles

The cooked corn noodles were freeze-dried and ground into a powder. The powder was mixed with potassium bromide at a ratio of 1:100 and further ground. The mixture was scanned in the wavelength range of 400–4000 cm^−1^ using Fourier-Transform Infrared Spectroscopy (FTIR) (VERTEX 70 FTIR, Bruker Inc., Billerica, Massachusetts, Germany). The obtained spectra were deconvoluted using OMNIC 8.2 software. The ratio of double helix structures (995 cm^−1^/1022 cm^−1^) and the ratio of short-range ordered structures (1047 cm^−1^/1022 cm^−1^) were then calculated.

### 2.15. Determination of Hydration Characteristics

The effects of different MEPS proportions on the solubility and swelling power of corn noodles were measured according to the method described by Zheng et al. [23]. The mixed powder was mixed with distilled water at a ratio of 1:5 (*w*/*v*). The mixture was thoroughly stirred and then stood at 4 °C for 10 min. Subsequently, it was centrifuged at 15,000 *g* for 30 min. *m*_1_, *m*_2_, and *m* correspond to the following measurements: *m*_1_ (mass of soluble fraction): The supernatant was collected, transferred to an aluminum dish, and dried to constant weight. *m*_2_ (mass of sediment): The mass of the precipitate collected in the centrifuge tube after centrifugation. *m* (initial dry mass of sample): The dry weight of the sample before extraction, on a dry-weight basis. Then, the hydration properties (solubility and swelling power) were calculated via the following formulas:

(6) $$\mathrm{Swelling}\;\mathrm{power}\;(g/g)\;=\;\frac{m_{2}}{m}$$

(7) $$\mathrm{Solubility}\;(\%)=\frac{m_{1}}{m}\times 100\%$$

### 2.16. Determination of Sulfhydryl (-SH) and Disulfide Bond (-SS) Content

The sulfhydryl content and disulfide bond content were determined according to the method described by Liu et al. [24]. The calculation formulas are as follows.

(8) $$\mathrm{Sulfhydryl}\;\mathrm{content}\;(\text{-}\mathrm{SH},\;\mathrm{\mu mol}/g)=\frac{73.53\;\times \;A_{412}\;\times \;D}{C}\;\times \;100\%$$

(9) $$\mathrm{Disulfide}\;\mathrm{bond}\;\mathrm{content}\;(\text{-}\mathrm{SS},\;\mathrm{\mu mol}/g)=\frac{\mathrm{Total}\;\mathrm{sulfhydryl}\;\mathrm{content}\;-\;\mathrm{Free}\;\mathrm{sulfhydryl}\;\mathrm{content}}{2}$$

A_412_ is the absorbance value at 412 nm, D is the dilution factor (6.04), and ρ is the protein concentration (mg/mL).

### 2.17. Analysis of Thermal Stability of Corn Noodles

The cooked corn noodles were freeze-dried and ground into a powder. Five milligrams of the freeze-dried powder were placed in a sample pan. The thermal stability of the sample was measured using a thermal gravimetric analyzer (Thermal Gravimetric Analyzer, METTLER-TOLEDO INTERNATIONAL Inc., Greifensee, Switzerland). Nitrogen was used as the carrier gas. The temperature was increased from 30 °C to 600 °C at a rate of 10 °C/min.

### 2.18. Statistical Analysis

All data were expressed as Mean ± SD. Statistical analysis was performed using one-way ANOVA. A *p*-value < 0.05 was considered statistically significant. Graphs were plotted using Prism 10 and Origin Pro 2021.

## 3. Results and Discussion

### 3.1. Effect of MEPS Addition on Dough Rheology and Water Distribution of Corn Dough

The main components of MEPS determine its potential functionality (Table 2). The total sugar content was about 43.2%, and the uronic acid content was about 15.2%. These results suggest that MEPS is a crude polysaccharide of edible mushroom rich in uronic acid, which helps to enhance the water-binding ability and biological activity of corn flour [25].

Gelatinization characteristics reflect the swelling and disruption of starch granules during heating, which directly affect dough handling and noodle texture [26]. The effect of MEPS on the gelatinization characteristics of corn flour is shown in Table 3. As the MEPS concentration increased from 0 to 0.6%, the peak viscosity progressively increased, reaching a maximum at 0.6%, and then slightly decreased at an MEPS concentration of 0.8% and 1.0%. The increase in peak viscosity is beneficial for noodle texture, as it contributes to better consistency and firmness [12]. It indicates that a moderate addition of MEPS promotes starch granule swelling and water absorption, likely through hydrogen bonding interactions between MEPS and starch molecules [27]. However, excessive MEPS may compete for water and physically coat starch granules, thereby restricting their expansion. The similar phenomenon occurred after adding oat β-glucan to rice starch according to the study by Li et al. [28].

In contrast to peak viscosity, the addition of MEPS significantly reduced breakdown, final viscosity, and setback values, with the most pronounced effects observed at 0.6% MEPS. Breakdown reflects the resistance of starch granules to mechanical shear during heating [29]. The lower breakdown values in MEPS-containing samples indicate that MEPS stabilizes the starch granule structure, reducing disintegration. Zhang et al. found that the addition of tremella polysaccharide to CS starch significantly increased the peak temperature and viscosity and reduced the degree of starch gelatinization [30]. This enhanced thermal stability is expected to lower cooking loss and improve the integrity of the noodles [31]. Final viscosity and setback are indicators of starch retrogradation [32]. The marked decreases observed suggest that MEPS interferes with the reassociation of amylose chains during cooling, likely via hydrogen bonding or encapsulation [12]. A lower setback implies a reduced tendency for starch recrystallization, which helps maintain noodle softness during storage and delays staling [33]. The pasting temperature showed only minor changes. A significant decrease from 80.2 °C to 78.8 °C was observed only at 0.8% MEPS, suggesting that high concentrations of MEPS may slightly disrupt the crystalline structure of starch, lowering the energy barrier for gelatinization. However, the overall effect on pasting temperature was limited. Collectively, the pasting results demonstrate that MEPS at optimal concentrations (0.4–0.6%) improves the water-binding, thermal stability, and anti-retrogradation ability of corn flour, all of which are favorable for the processing quality of corn noodles.

**Figure 1:**
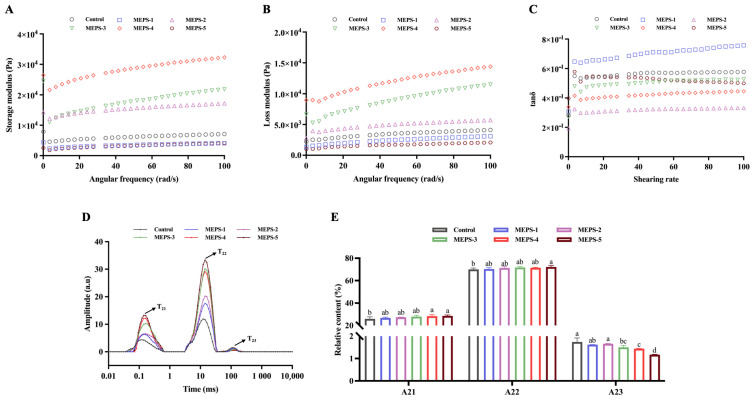
Effects of MEPS addition amount on (**A**) storage modulus (G’), (**B**) loss modulus (G’’), tanδ (**C**) and water distribution (**D**,**E**) of corn dough. Note: Different letters represent significant differences between different groups (*p* < 0.05).

To further understand how MEPS affects dough viscoelasticity, dynamic rheological measurements were performed. The results are shown in Figure 1A–C. Compared to the control group, adding MEPS-1 (0.2%) and MEPS-5 (1.0%) led to a decrease in both G’ and G’’. Conversely, MEPS-2, MEPS-3, and MEPS-4 significantly increased both G’ and G’’. This indicates that 0.4–0.8% MEPS reinforces the three-dimensional network structure of the dough, enhancing its rigidity and resistance to deformation. The weakening effect observed at MEPS-1 and MEPS-5 suggests that insufficient polysaccharides fail to form an effective network, while excessive amounts may oversaturate the system and physically hinder protein–starch interactions, leading to a less structured dough matrix [12]. The tanδ reflects the relative contribution of viscosity to elastic behavior. Compared to the control, only adding MEPS-1 increased the tanδ value, indicating a more fluid-like, less elastic dough. In all other groups, tanδ values were significantly lower than the control. It demonstrated that adding suitable polysaccharides could transform the dough into a more elastic system [34]. The elastic reinforcement could improve dough handling during sheeting, reduce stickiness, and contribute to a firmer, more resilient noodle texture after cooking [35].

The improved viscoelasticity is often associated with changes in water distribution. Therefore, LF-NMR was used to evaluate water mobility and binding states. The water distribution measured by LF-NMR is shown in Figure 1D,E. The T_2_ relaxation spectra exhibited three distinct peaks (T_21_, T_22_, T_23_), corresponding to bound water, immobilized water, and free water, respectively. Compared with the control group, the addition of MEPSs caused a progressive increase in the proportions of A_21_ and A_22_ as the MEPS concentration increased. In contrast, the proportion of A_23_ decreased significantly in a dose-dependent manner. These results indicated that MEPS enhances the water-holding capacity of corn dough. The increase in bound water (A_21_) suggests that MEPS, through its abundant hydroxyl groups, forms hydrogen bonds with water molecules, thereby immobilizing water at the molecular level. The rise in immobilized water (A_22_) reflects the entrapment of water within the three-dimensional network formed by MEPSs and starch/protein components. Conversely, the reduction in free water (A_23_) implies that less water is available for free movement, which is often associated with a more compact and stable dough structure. From a processing perspective, the MEPS-induced redistribution of water from a free to a more bound or immobilized state is beneficial for noodle quality. A higher proportion of bound and immobilized water reduces water loss during sheeting and cooking, which helps lower cooking loss and improves noodle integrity [36]. Moreover, the enhanced water retention contributes to a softer yet firmer texture by maintaining adequate moisture within the starch–protein network [31].

### 3.2. Effect of MEPS Addition on the Cooking Process of Corn Noodles

The above results demonstrated that the addition of MEPS improved the characteristics of corn dough. Therefore, the study further evaluated the cooking quality of noodles, including water absorption, breakage rate and cooking loss. Compared with the control group, the water absorption of corn noodles increased significantly from MEPS-2 to MEPS5, while MEPS-1 showed no significant change. This concentration-dependent increase suggests that MEPS enhances the water-holding capacity of the noodle matrix. The abundant hydroxyl groups in MEPS form hydrogen bonds with water molecules, while the polysaccharide network physically entraps water within the starch–protein structure, which is similar to the trend of water distribution change [31]. Higher water absorption is generally associated with a softer and more palatable texture, which is desirable for consumer acceptance. The breakage rate of corn noodles decreased significantly from MEPS-2 to MEPS-4, whereas no significant effect was observed at MEPS-1 and MEPS-5. The reduction in breakage rate at moderate MEPS levels indicates that the polysaccharide reinforces the noodle structure, making it more resistant to fracture during cooking. This reinforcement is likely due to the formation of a continuous polysaccharide–starch/protein network that holds the noodle together. MEPS-1 may be insufficient to build an effective network. MEPS-5 may cause local over-concentration or interfere with starch gelatinization, thereby negating the beneficial effect on structural integrity, which is similar to previous studies; the processing properties of mushroom polysaccharides on starch are related to concentration [37]. Cooking loss, which reflects the amount of solid leached from noodles into the cooking water, was significantly reduced at all MEPS concentrations except 0.4%. The largest reductions were observed at 0.6–1.0% MEPS. A lower cooking loss indicates that MEPS effectively retains starch and other components within the noodle matrix. This is achieved through the physical encapsulation of starch granules by the polysaccharide network and enhanced hydrogen bonding interactions, which reduce the leaching of soluble materials. In summary, the addition of MEPS at suitable concentrations significantly improves the overall cooking quality of corn noodles by increasing water absorption, reducing breakage rate, and decreasing cooking loss. These positive effects are attributed to the water-binding capacity and network-forming ability of MEPS.

**Figure 2:**
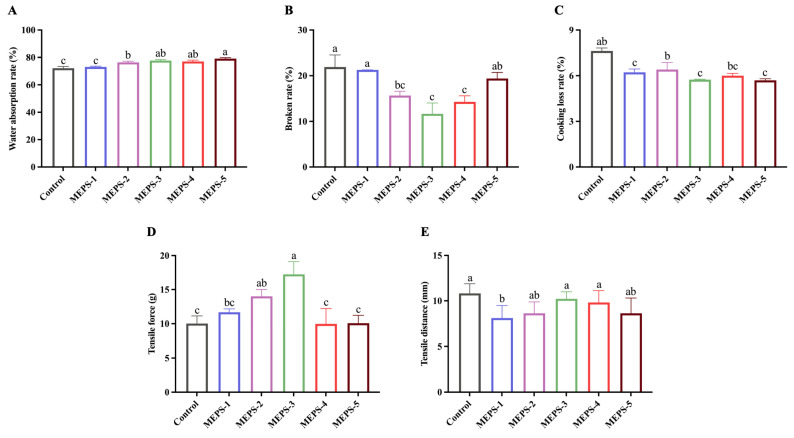
Effects of MEPS addition amount on steaming and boiling quality (**A**–**C**) and tensile properties (**D**,**E**) of corn noodles. Note: Different letters represent significant differences between different groups (*p* < 0.05).

To evaluate the mechanical strength of corn noodles, the tensile properties, including breaking force and extension distance, were measured to evaluate their resistance to rupture and deformability. The tensile properties of corn noodles are presented in Figure 2D,E. Compared with the control, MEPS-2 and MEPS-3 significantly increased the breaking force, but other groups showed no significant change. This indicates that a moderate amount of MEPS effectively strengthens the noodle matrix, while both insufficient and excessive polysaccharide addition fail to provide a reinforcing effect. The increase in breaking force at 0.4–0.6% MEPSs is attributed to the enhanced network crosslinking formed by hydrogen bonding and physical entanglement between MEPS and starch/protein components [31]. This network increases the mechanical integrity of noodles, making them more resistant to breakage during handling and consumption. The extension distance was significantly reduced only at 0.2% MEPS, with no significant changes observed at other concentrations. A shorter extension distance suggests reduced extensibility, which may be caused by a discontinuous or weakly formed network that breaks more easily under tension [38]. At higher MEPS levels (0.4–1.0%), the network appears to be sufficiently developed to maintain normal extensibility without compromising flexibility. Collectively, the optimal improvement in tensile properties is achieved at 0.4–0.6% MEPSs, where both breaking force and extension distance are maintained or enhanced. This concentration range contributes to a firmer yet sufficiently extensible noodle structure.

Furthermore, the textural properties of corn noodles, including hardness, springiness, adhesiveness, and resilience, were significantly affected by MEPS addition. The texture properties of corn noodles are presented in Table 4. Hardness increased progressively with MEPS concentration. This dose-dependent reinforcement indicates that MEPS strengthens the noodle matrix. The strengthening effect is attributed to hydrogen bonding and physical entanglement between MEPSs and starch/protein components, which form a more resilient three-dimensional network. Springiness showed a non-linear response. At 0.4% and 0.6% MEPS, springiness remained comparable to the control. In contrast, at 0.2%, 0.8%, and 1.0% MEPS, springiness decreased significantly. This suggests an optimal concentration range (0.4–0.6%) for preserving the elastic recovery of noodles. Insufficient MEPS fails to form an effective network, while excessive amounts may oversaturate the system and restrict polymer chain mobility, thereby reducing springiness [39]. All MEPS concentrations significantly reduced adhesion, which was beneficial to prevent noodle caking and improve palatability. The reduction is explained by the high water-binding capacity of MEPS, which diminishes free water on the noodle surface, and by the formation of a continuous polysaccharide film that minimizes surface tackiness. Resilience was slightly decreased at 0.2% MEPS, but no significant differences were observed at higher concentrations (0.4–1.0%) compared with the control. Thus, MEPS does not adversely affect the speed of elastic recovery. Collectively, MEPS at 0.4–0.6% optimally improves the textural quality of corn noodles by increasing hardness, maintaining springiness, reducing adhesiveness, and preserving resilience.

### 3.3. Effect of MEPS Addition on Starch Digestibility and Estimated Glycemic Index of Corn Noodles

**Figure 3:**
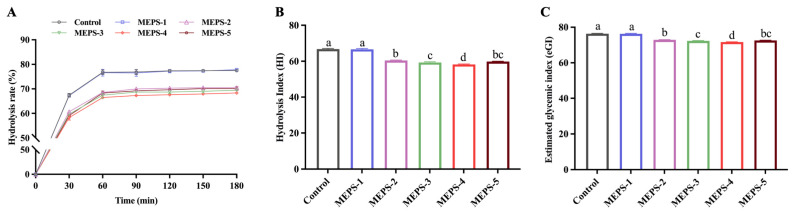
Effects of MEPS addition amount on hydrolysis rate (**A**), HI (**B**), and eGI (**C**) of corn noodles. Note: Different letters represent significant differences between different groups (*p* < 0.05).

To investigate whether the improved processing quality of MEPS-enriched corn noodles affects starch digestion, in vitro starch hydrolysis curves were determined. The results are shown in Figure 3A. Compared with the control group, the addition of MEPS at 0.4–1.0% significantly reduced the HI and the eGI. In these groups, the hydrolysis rate was notably slower during the first 60 min of digestion, and the final hydrolysis extent was lower. In contrast, the 0.2% MEPS group showed no significant difference from the control in either HI or eGI. The reduction in HI and eGI at 0.4–1.0% MEPS indicates that the polysaccharide effectively delays starch digestion. This effect is attributed to the physical barrier formed by the MEPS network, which encapsulates starch granules and limits enzyme accessibility [40]. The slow digestion rate during the initial 60 min is particularly important, as this period corresponds to the rapid hydrolysis of readily digestible starch. The lack of effect at 0.2% MEPS suggests that this concentration is insufficient to create a continuous protective network, which is consistent with the limited improvements in processing quality observed at the same concentration. Moreover, compared with control group, the eGI values of MEPS-2 to MEPS-5 significantly decreased. This shift confirms that adding MEPSs is beneficial for postprandial blood glucose management of corn noodles. Collectively, the starch digestibility results demonstrate that 0.4–1.0% MEPS significantly lowered the predicted glycemic response of corn noodles. The effect is consistent with the structural reinforcement observed in the processing quality analysis, further supporting the role of MEPS in forming a resistant network that retards starch hydrolysis.

### 3.4. Effect of MEPS Addition on Antioxidant and Enzyme Inhibitory Activities of Corn Noodles

**Figure 4:**
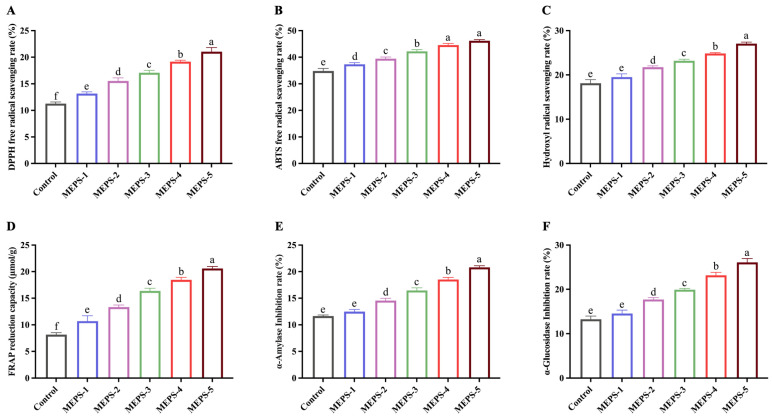
Effects of MEPS addition amount on DPPH scavenging capacity (**A**), ABTS scavenging capacity (**B**), hydroxyl radical-scavenging capacity (**C**), FRAP ability (**D**), α-amylase (**E**) and α-glucosidase (**F**) inhibition rate of corn noodles. Note: Different letters represent significant differences between different groups (*p* < 0.05).

In addition to the improvement of processing characteristics, corn noodles are endowed with bioactive effects by MEPSs. With increasing MEPS concentration, the DPPH radical-scavenging activity, ABTS radical-scavenging activity, FRAP value, hydroxyl radical scavenging activity, α-amylase inhibition, and α-glucosidase inhibition all increased significantly (Figure 4). This dose-dependent enhancement indicates that MEPS effectively transfers its bioactive properties to the noodle matrix, likely through the retention of polysaccharide chains that remain active after cooking. At 0.4–1.0% MEPS, all six bioactivity indicators were significantly elevated. The simultaneous enhancement of antioxidant and enzyme inhibitory activities at these concentrations adds functional value to the corn noodles beyond their improved processing quality. In particular, the inhibition of α-glucosidase and α-amylase provides a biochemical mechanism that complements the physical barrier effect and explains the reduced starch hydrolysis rate and eGI [41]. However, adding 0.2% MEPS exhibited a distinct pattern. At this concentration, DPPH scavenging, ABTS scavenging, and FRAP were significantly improved compared with the control, whereas hydroxyl radical scavenging, α-amylase inhibition, and α-glucosidase inhibition showed no significant change. This differential response suggests that lower concentrations of MEPSs are sufficient to mediate electron-transfer-based antioxidant activities (DPPH, ABTS, FRAP) but are insufficient to directly quench hydroxyl radicals or inhibit the two key digestive enzymes. The lack of enzyme inhibition at 0.2% MEPS is consistent with the earlier observation that this concentration did not reduce starch digestibility, further supporting the link between enzyme inhibition and the lowering of eGI. In summary, MEPS at 0.4–1.0% effectively improves both the antioxidant capacity and the hypoglycemic enzyme inhibitory activities of corn noodles, while 0.2% MEPS only enhances certain radical-scavenging properties.

### 3.5. Effect of MEPS Addition on Microstructure and Molecular Interactions of Corn Noodles

Based on the comprehensive evaluation of processing quality and functional properties, two representative MEPS concentrations (0.4% and 0.6%) were selected for mechanistic investigation.

**Figure 5:**
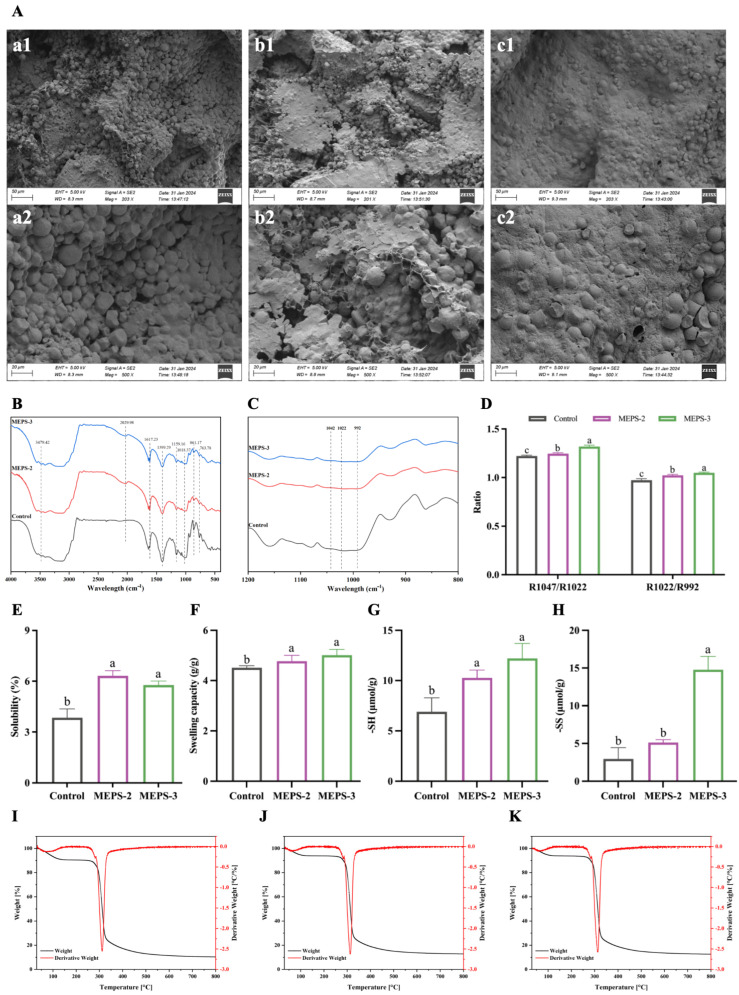
Effects of MEPS addition on the microstructure (**A**) (Microstructure of control groups (**a1**,**a2**), MEPS-2 (**b1**,**b2**), and MEPS-3 (**c1**,**c2**)), secondary structure (**B**–**D**), hydrogenation characteristics (**E**,**F**), sulfhydryl content (**G**), disulfide bond content (**H**) and thermal stability (**I**–**K**) of corn noodles. Note: Different letters represent significant differences between different groups (*p* < 0.05).

The microstructure of corn noodles from the control, MEPS-2, and MEPS-3 groups was observed by SEM. The images are shown in Figure 5A. The control group exhibited large gaps between the granules, indicating a loose and discontinuous structure with limited interactions among starch and protein components. This loose structure explains the poor processing quality of control noodles, such as the high cooking loss and breakage rate. When 0.4% MEPS was added, a loose noodle mesh appeared between the granules. Some granules remained exposed on the surface of the protein network. This partial coverage suggests that 0.4% MEPS begins to form intermolecular associations with starch and protein, but the network is not yet fully developed. This observation is consistent with the intermediate improvements in cooking and textural properties at 0.4% MEPS, such as reduced breakage rate and increased hardness, but without significant change in springiness or cooking loss. At 0.6% MEPS, a marked increase in network density was observed. Most granules were embedded within a continuous gel network, and the structure exhibited good continuity and compactness. This well-developed network indicates that 0.6% MEPS effectively promotes crosslinking among starch granules and protein components, likely through hydrogen bonding and physical entanglement. The encapsulation of starch granules by the polysaccharide network provides a physical barrier that restricts enzyme accessibility, which directly explains the reduced starch hydrolysis rate and lower eGI [37]. Furthermore, the dense and continuous structure accounts for the significantly improved cooking quality and enhanced textural properties at this concentration.

To elucidate the possible interaction between MEPS and protein and starch in corn flour, the FTIR spectra of corn noodles were analyzed. As is shown in Figure 5B, no significant shift in absorption peak positions was observed, and no new characteristic peaks appeared after MEPS addition. It indicated that MEPS does not form covalent bonds with starch or protein components; instead, the interactions are predominantly physical. Moreover, the ratios of R_1047/1022_ and R_1022/995_ were calculated to evaluate changes in the short-range molecular order and double helix structure of starch. As is shown in Figure 5C,D, R_1047/1022_ and R_1022/995_ increased significantly with increasing MEPS concentration. The R_1047/1022_ of MEPS-3 reached 1.319 ± 0.014, and the R_1022/995_ reached 1.049 ± 0.008. These increases indicate that the addition of MEPS promotes molecular rearrangement of starch in the noodle matrix. The higher R_1047/1022_ ratio reflects an increase in short-range ordered structures, while the higher R_1022/995_ ratio suggests an enhanced proportion of double helices. Both changes imply stronger intermolecular forces (mainly hydrogen bonding) among starch chains and between starch and MEPS [42]. Consequently, a more ordered gel network is formed, which is consistent with the compact and continuous microstructure observed by SEM. This improved molecular ordering provides a structural basis for the enhanced processing quality of MEPS-enriched corn noodles. Specifically, the denser and more ordered network restricts granule swelling and leaching during cooking, leading to reduced cooking loss and improved hardness. At the same time, the physical barrier created by the ordered network limits enzyme access to starch, contributing to the lower starch hydrolysis rate and eGI.

The −SH and −SS− contents, as well as the solubility and swelling capacity of corn noodles, were examined to further understand the molecular interactions induced by MEPS. MEPS-3 significantly increased both the −SH and −SS− contents compared with the control. In contrast, MEPS-2 only significantly increased the −SS− content, with no significant change in −SH. The increase in −SS− at both concentrations indicates that MEPS promotes the formation of disulfide bonds, likely through the rearrangement of protein conformations or by creating an oxidative environment that favors −SH oxidation [43]. The additional increase in −SH at 0.6% MEPS suggests that at this higher concentration, MEPS may also expose buried -SH groups, possibly by unfolding protein chains via hydrogen bond competition. Enhanced disulfide crosslinking strengthens the protein network within the noodle matrix, which contributes to the improved hardness, reduced cooking loss, and lower breakage rate [44]. Compared with the control, both the solubility and swelling capacity of corn noodles increased significantly after MEPS addition. Greater solubility indicates that more soluble polysaccharides and small molecular components can be released during hydration, which may be related to the improved water absorption and reduced adhesiveness observed earlier. Enhanced swelling capacity reflects the ability of the MEPS–starch/protein network to imbibe and retain water. This is consistent with the increased water absorption and the higher proportion of bound and immobilized water measured by LF-NMR. The improved hydration properties further support the role of MEPS as a water-binding and network-stabilizing agent. Together, the increased disulfide crosslinking and enhanced hydration properties provide complementary molecular-level explanations for the superior processing quality and functional performance of MEPS-enriched corn noodles, particularly MEPS-3.

The thermal degradation behavior of corn flour mixed with different MEPS concentrations was evaluated by TGA. All samples exhibited two major stages of mass loss. The first stage occurred between 30 °C and 180 °C and was mainly attributed to water evaporation. The second stage occurred at higher temperatures and was characterized by a faster rate of mass loss, primarily due to the thermal decomposition of starch and MEPS. In first stage, the control group lost 9.5187% of its initial mass, whereas MEPS-2 lost 6.1835%, and MEPS-3 lost 6.1002%. The lower mass loss in MEPS groups indicated that MEPS reduces the content of free water, which is consistent with the increased bound-water proportion observed by LF-NMR. This water-binding effect contributes to the enhanced water-holding capacity of the dough and noodles. In the second stage, the control group lost 81.2356% of its mass, MEPS-2 lost 81.0555%, and MEPS-3 lost 80.1540%. Compared with the control, the MEPS-3 (adding 0.6% MEPS) showed the lowest mass loss in the second stage, indicating that MEPS interferes with the thermal stability of starch. Specifically, the presence of MEPS shifts the degradation to a slightly higher temperature or reduces the extent of decomposition, which suggests enhanced thermal stability of the gel network [45].

## 4. Conclusions

In summary, adding 0.6% MEPS significantly improved both the processing quality and bioactive properties of corn noodles. At this concentration, MEPS enhanced dough viscoelasticity and water-holding capacity, increased peak viscosity, reduced breakdown and setback, and promoted the formation of a compact, continuous gel network with increased molecular order and disulfide crosslinking. Consequently, cooking loss and breakage rate decreased, while hardness increased and adhesiveness was reduced without compromising springiness. Moreover, the well-developed network physically encapsulated starch granules and, together with the direct inhibition of α-amylase and α-glucosidase, markedly slowed starch hydrolysis during the initial 60 min of digestion, lowering the estimated glycemic index from a high to a medium range. Meanwhile, MEPS endowed the noodles with dose-dependent antioxidant activities, including DPPH, ABTS, hydroxyl radical scavenging, and FRAP. These findings demonstrate that MEPS is a natural, multifunctional ingredient that simultaneously upgrades the processing quality of corn noodles and confers significant health-promoting functions. This work provides a new strategy to improve the quality of cornmeal processing, offering new theoretical and practical foundations to promote the staple food application of corn.

## Figures and Tables

**Table 1:** Grouping and composition of corn noodles.

Sample	Corn Flour (g)	Vital Gluten (g)	Salt (g)	MEPS (g)	Purified Water (g)
Control	100	14	1	0	60
MEPS-1	100	14	1	0.2	60
MEPS-2	100	14	1	0.4	60
MEPS-3	100	14	1	0.6	60
MEPS-4	100	14	1	0.8	60
MEPS-5	100	14	1	1	60

**Table 2:** The main components of MEPS.

Sample	Total Sugar Content (%)	Uronic Acid Content (%)	Protein Content (%)
MEPS	43.24 ± 0.51	15.17 ± 0.81	1.17 ± 0.03

**Table 3:** Effects of MEPS addition amount on gelatinization characteristics of corn flour.

Sample	Peak Viscosity (cP)	Final Viscosity (cP)	Breakdown (cP)	Setback (cP)	Pasting Temperature (°C)
Control	1591.000 ± 17.349 ^b^	2691.667 ± 19.743 ^a^	469.000 ± 16.042 ^a^	1465.333 ± 19.616 ^a^	80.233 ± 0.259 ^ab^
MEPS-1	1604.333 ± 43.129 ^ab^	2606.667 ± 28.812 ^d^	435.667 ± 47.792 ^b^	1411.333 ± 50.719 ^ab^	79.617 ± 0.259 ^ab^
MEPS-2	1670.667 ± 48.875 ^ab^	2624.667 ± 19.428 ^c^	391.000 ± 36.756 ^e^	1369.667 ± 18.342 ^ab^	79.617 ± 0.285 ^ab^
MEPS-3	1695.333 ± 17.072 ^a^	2544.000 ± 70.995 ^f^	388.333 ± 41.168 ^f^	1328.000 ± 24.111 ^b^	79.633 ± 0.934 ^ab^
MEPS-4	1646.000 ± 21.939 ^ab^	2544.667 ± 34.854 ^e^	392.000 ± 48.446 ^d^	1345.667 ± 41.842 ^b^	78.800 ± 0.301 ^b^
MEPS-5	1631.000 ± 16.503 ^ab^	2639.000 ± 83.722 ^b^	412.333 ± 27.168 ^c^	1380.667 ± 42.306 ^ab^	80.983 ± 0.517 ^a^

Note: Different letters on the same column represent significant differences between different groups (*p* < 0.05).

**Table 4:** Effects of MEPS addition amount on the texture characteristics of corn noodles.

Sample	Hardness/g	Springiness	Cohesiveness	Resilience
Control	3062.80 ± 77.33 ^f^	0.93 ± 0.05 ^ab^	57.00 ± 4.24 ^f^	0.27 ± 0.01 ^ab^
MEPS-1	3354.40 ± 423.12 ^e^	0.88 ± 0.06 ^bc^	55.44 ± 2.39 ^d^	0.25 ± 0.02 ^b^
MEPS-2	3527.93 ± 166.21 ^d^	0.92 ± 0.02 ^ab^	49.35 ± 3.54 ^b^	0.28 ± 0.01 ^a^
MEPS-3	3787.00 ± 166.72 ^c^	0.97 ± 0.04 ^a^	45.96 ± 4.89 ^a^	0.30 ± 0.01 ^a^
MEPS-4	3879.71 ± 415.55 ^b^	0.87 ± 0.03 ^bc^	51.27 ± 17.97 ^c^	0.30 ± 0.02 ^a^
MEPS-5	3952.67 ± 136.87 ^a^	0.84 ± 0.03 ^c^	47.64 ± 6.76 ^e^	0.30 ± 0.01 ^ab^

Note: Different letters on the same column represent significant differences between different groups (*p* < 0.05).

## Data Availability

The original contributions presented in the study are included in the article, further inquiries can be directed to the corresponding authors.

## Abbreviations

The following abbreviations are used in this manuscript:

MEPSs	Magnetically treated *Ganoderma lucidum* extracellular polysaccharides
RDS	Rapidly digestible starch
eGI	Estimated glycemic index
RVA	Rapid visco analyzer
G’	Storage modulus
G’’	Loss modulus
tanδ	Loss factor
LF-NMR	Low-field nuclear magnetic resonance
DNS	3,5-dinitrosalicylic acid
AUC	Area under the hydrolysis curve
HI	Hydrolysis index
FRAP	Ferric reducing antioxidant power
SEM	Scanning electron microscope
FTIR	Fourier-transform infrared spectroscopy
-SH	Sulfhydryl
-SS	Disulfide bond
